# *In Vitro* Antifungal Activity of Sertraline and Synergistic Effects in Combination with Antifungal Drugs against Planktonic Forms and Biofilms of Clinical *Trichosporon asahii* Isolates

**DOI:** 10.1371/journal.pone.0167903

**Published:** 2016-12-08

**Authors:** Lin Cong, Yong Liao, Suteng Yang, Rongya Yang

**Affiliations:** 1 Graduate School, Third Military Medical University, chongqing, China; 2 Department of Dermatology, PLA Army General Hospital, Beijing, China; University of Minnesota, UNITED STATES

## Abstract

*Trichosporon asahii* (*T*. *asahii*) is the major pathogen of invasive trichosporonosis which occurred mostly in immunocompromised patients. The biofilms formation ability of *T*. *asahii* may account for resistance to antifungal drugs and results a high mortality rate. Sertraline, a commonly prescribed antidepressant, has been demonstrated to show *in vitro* and *in vivo* antifungal activities against many kinds of pathogenic fungi, especially *Cryptococcus* species. In the present study, the *in vitro* activities of sertraline alone or combined with fluconazole, voriconazole, itraconazole, caspofungin and amphotericin B against planktonic forms and biofilms of 21 clinical *T*. *asahii* isolates were evaluated using broth microdilution checkerboard method and XTT reduction assay, respectively. The fractional inhibitory concentration index (FICI) was used to interpret drug interactions. Sertraline alone exhibited antifungal activities against both *T*. *asahii* planktonic cells (MICs, 4–8 μg/ml) and *T*. *asahii* biofilms (SMICs, 16–32 μg/ml). Furthermore, SRT exhibited synergistic effects against *T*. *asahii* planktonic cells in combination with amphotericin B, caspofungin or fluconazole (FICI≤0.5) and exhibited synergistic effects against *T*. *asahii* biofilms in combination with amphotericin B (FICI≤0.5). SRT exhibited mostly indifferent interactions against *T*. *asahii* biofilms in combination with three azoles in this study. Sertraline-amphotericin B combination showed the highest percentage of synergistic effects against both *T*. *asahii* planktonic cells (90.5%) and *T*. *asahii* biofilms (81.0%). No antagonistic interaction was observed. Our study suggests the therapeutic potential of sertraline against invasive *T*. *asahii* infection, especially catheter-related *T*. *asahii* infection. Further *in vivo* studies are needed to validate our findings.

## Introduction

*Trichosporon asahii* (*T*. *asahii*) is an opportunistic pathogen which belongs to the member of basidiomycete yeast-like fungi and can cause invasive trichosporonosis in immunocompromised patients [1].

The incidence of invasive trichosporonosis has been increased over the past 4 decades with the increased immunocompromised population, mainly those with hematological malignant diseases, AIDS patients and organ transplant recipients [2]. Additional risk factors include the use of corticosteroid, chemotherapy, as well as the use of medical implanted devices [2].

Various antifungal drugs have been used in the treatment of invasive trichosporonosis, including polyenes (such as amphotericin B), echinocandins (such as caspofungin) and the azoles (such as fluconazole, itraconazole and voriconazole). However, *T*. *asahii* often cause breakthrough infections in patients treated with AMB or echinocandins [3–5].

In a clinical guideline for the diagnosis and management of rare invasive yeast infection (including *Trichosporon* species), amphotericin B monotherapy is not recommended for invasive trichosporonosis, because of its limited *in vitro* activity against *T*. *asahii* (MICs≥2 mg/L) and poor response rates (between 16% and 24%) to trichosporonosis [6]. Echinocandins are also not recommended for treating invasive trichosporonosis since *Trichosporon* spp. is intrinsic resistant to this antifungal drug class [1,6].

The newer triazoles (such as voriconazole) are now considered to be the most effective drugs class for invasive trichosporonosis treatment because they exhibit good *in vitro* and *in vivo* activity against *Trichosporon* spp. and result good clinical outcome [1,6]. However, the high cost of new triazoles impedes their widespread use in China. Furthermore, since azoles are all fungistatic, the sustained use of azoles antifungal drugs may result in drug-resistance, especially when used as low-dose prophylactic/empirical therapy. Actually, decreased susceptibility of *T*. *asahii* to azoles has been reported and multidrug-resistant *Trichosporon* strains have already been isolated [7,8].

Invasive *T*. *asahii* infections are usually associated with the use of medical implanted devices (such as central venous catheters, vesical catheters, and peritoneal catheter-related devices) [1]. The ability of *T*. *asahii* to form biofilms on medical implanted devices may account for the clinical resistance to antifungal drugs and results a high mortality rate. Although the newer triazoles have been demonstrated to show excellent *in vitro* activity against *T*. *asahii* planktonic cells, they have been reported failing to eradicate *T*. *asahii* biofilms and may result in treatment failure [9,10]. Thus, in the views of drug-resistance and pharmacoeconomics, it is necessary to develop new therapeutic approach against *T*. *asahii* infection. To our knowledge, combination of traditional antifungal drugs with non-antifungal agents has been proposed to be a promising strategy to cope with resistant fungal infections [11,12], this antifungal strategy may also be beneficial to cope with resistant *T*. *asahii* infections.

Sertraline (SRT) is a commonly prescribed antidepressant that belongs to the group of selective serotonin reuptake inhibitors [13]. It has been demonstrated that SRT exhibit antifungal activities against *Candida* spp., *Aspergillus* spp. and *Cryptococcus* species [14–18]. SRT has also been demonstrated to show *in vitro* synergistic effects in combination with antifungal drugs against *Aspergillus* spp. and *Cryptococcus neoformans* (*C*. *neoformans*) [19–21]. Furthermore, SRT was demonstrated to exhibit adjunctive antifungal effect against HIV-associated cryptococcal meningitis clinically [22]. Considering that *Trichosporon* spp. is phylogenetically closed to *Cryptococcus* species, we wonder if SRT has similar antifungal activity and synergistic effect against *T*. *asahii*. To our knowledge, no studies have been conducted on the antifungal activity of SRT against *Trichosporon* species.

In the present study, the *in vitro* antifungal activities of SRT alone or in combination with clinical commonly used antifungal drugs against planktonic forms of 21 clinical *T*. *asahii* isolates were examined by a broth microdilution checkerboard method based on M27-A3 reference method documented by Clinical and Laboratory Standards Institute (CLSI) [23]. *In vitro* anti-biofilms activities of SRT alone or in combination with antifungal drugs were examined by a XTT reduction assay. The results of our *in vitro* antifungal susceptibility testing against *T*. *asahii* may be helpful to evaluate the possible application of SRT in treating *T*. *asahii* infections.

## Materials and Methods

### Fungal Strains

A total of 21 clinical isolates of *T*. *asahii* were used in this study. The clinical type strain of *T*. *asahii* (CBS2479) was purchased from the CBS-KNAW Fungal Biodiversity Centre (the Netherlands). Sixteen clinical strains (BZP07001, BZP07002, BZP07003, BZP07004, BZP07005, BZP07006, BZP07007, BZP07008, BZP07009, BZP07010, BZP07011, BZP07012, BZP07013, BZP07014, BZP09001, BZP09002) were collected from patients in PLA Army General Hospital (Beijing, China) over a period of 12 years from 2003 and 2015. Four clinical strains (6108, 6198, 6674, 6956) were kindly provided by Research Center for Medical Mycology, Peking University First Hospital (Beijing, China). These isolates were identified as *T*. *asahii* by using a commercial kit (API 20C AUX, BioMeorieux, France) and by DNA sequencing of the intergenic spacer 1 (IGS1, GenBank: AB066386.1) region of the rRNA gene.

All strains were removed from -80°C freezer, and then subcultured twice on Sabouraud dextrose agar (SDA, Merck KGaA, Darmstadt, Germany) at 35°C for 24 to 48 h to ensure purity and viability. The subcultures were further cultured overnight in yeast peptone dextrose (YPD, Oxoid Limited, England) liquid medium at 37°C in a rotating incubator at 130 rpm. Following growth, the cells were harvested by centrifugation and washed twice with sterile phosphate-buffered saline (PBS). The cells were resuspended in RPMI 1640 medium, which has been adjusted to pH 7.0 with 0.165 M morpholinepropanesulfonic acid (MOPS) (Sigma-Aldrich, St Louis, MO, USA) to the densities of 10^3^ CFU/ml for the *in vitro* susceptibility testing against planktonic cells and 10^6^ CFU/ml for the *in vitro* anti-biofilms susceptibility testing. *Candida parapsilosis* ATCC 22019 was included as quality control strain for our *in vitro* susceptibility testing.

### Drugs

Fluconazole (FLC), itraconazole (ITC), voriconazole (VRC), caspofungin (CAS), amphotericin B (AMB) and sertraline (SRT) were all purchased from Sigma-Aldrich (St. Louis, MO, USA). The stock solutions of ITC (100 mg/ml), VRC (100 mg/ml), AMB (100 mg/ml) and SRT (100 mg/ml) were freshly prepared in dimethyl sulfoxide (DMSO, Sigma). FLC (100 mg/ml) and CAS (100 mg/ml) were dissolved in sterile distilled water. After serial dilution, the final concentration of DMSO was below 1%.

### *In vitro* antifungal susceptibility testing against *T*. *asahii* planktonic cells

*In vitro* activities of FLC, ITC, VRC, CAS, AMB or SRT alone, and combinations of SRT with antifungal drugs against *T*. *asahii* planktonic cells were evaluated by using the broth microdilution checkerboard method based on the M27-A3 reference method (CLSI, USA) [23]. All tested drugs were distributed in 96-well microtitre plates. The final drug concentrations ranged from 0.062 to 64 μg/ml for FLC, from 0.008 to 8 μg/ml for ITC and AMB, from 0.001 to 1 μg/ml for VRC, from 0.031 to 32 μg/ml for CAS, and from 0.5 to 32 μg/ml for SRT. *T*. *asahii* cells suspensions were adjusted to a 0.5 McFarland standard transmittance at 530 nm wavelength. After that, the final inoculums of *T*. *asahii* were approximately 1.0×10^3^−3.0×10^3^ CFU/ml in each well after a serial dilution with RPMI 1640 broth medium. The plates were then incubated at 35°C for 48 h. Thereafter, the minimum inhibitory concentrations (MICs) were recorded according to M27-A3 guideline [23]. The MICs of FLC, ITC VRC and CAS were defined as 50% reduction in turbidity compared to the growth control wells and the MIC for AMB was defined as complete inhibition of growth. To investigate the possible fungicidal activity of SRT, both MIC-2 (50% reduction in turbidity compared to the growth control well) and MIC-0 (complete inhibition of growth) endpoints were used for SRT in this study. The MIC-2 endpoint was also used for AMB to allow the antifungal combinations susceptibility testing to be comparable between all tested drugs. The MICs that inhibited 50 and 90% of the total isolates were defined as MIC_50_ and MIC_90_, respectively. RPMI 1640 medium without *T*. *asahii* cells, as well as drug-free medium containing *T*. *asahii* cells, was used as negative and positive controls respectively. Experiments were repeated three times on different days.

### Biofilms formation of *T*. *asahii* and *in vitro* anti-biofilms susceptibility testing

The biofilms formation of *T*. *asahii* was performed by a simple and reproducible 96-well plates-based method as previously described [24]. Briefly, 100 μl adjusted *T*. *asahii* suspension (10^6^ CFU/ml) was added to 96-well plates. The wells containing RPMI 1640-MOPS medium without *T*. *asahii* cells were included as background controls. After 1 h of incubation (adhesion phase) at 37°C, each well was washed twice gently with sterile PBS to remove non-adherent cells, 200 μl of fresh RPMI 1640-MOPS medium was then added to each well and the plates were further incubated at 37°C for 24 h.

According to a protocol previously described [24], *in vitro* activities of FLC, ITC, VRC, AMB, CAS or SRT alone, and combinations of SRT with antifungal drugs against *T*. *asahii* biofilms were assessed by the 2,3-bis (2-methoxy-4-nitro-5-sulfophenyl)-5-[(phenylamino)-carbonyl]-2H-tetrazolim hydroxide (XTT) (Sigma-Aldrich, St. Louis, MO, USA) reduction assay [25]. After a 24 h incubation at 37°C to allow biofilms formation, the medium was replaced with fresh RPMI 1640 (pH 7.0-MOPS) supplemented with the antifungal drugs to the following final concentrations: FLC (2–1024 μg/ml), ITC (2–1024 μg/ml), VRC (2–1024 μg/ml), AMB (2–1024 μg/ml), CAS (0.062–64 μg/ml) and SRT (1–64 μg/ml), the plates were then incubated for another 24 h at 37°C. After that, the medium was replaced by 100 μl freshly prepared XTT/menadione solution in each well, the plates were incubated in the dark for 2 h at 37°C. Thereafter, 80 μl supernatant from each well was transferred to another microtiter plate, then the absorbance was read using a microplate reader (Thermo Fisher Scientific Inc., USA) at 492 nm.

According to previously studies [10,23], the sessile MICs (SMICs) were defined as the lowest concentration capable of decreasing 50% in absorbance compared to the growth control wells measured by XTT reduction assay. Experiments were repeated three times on different days.

### Drug interaction analysis

Drug combination interaction was evaluated on the basis of the fractional inhibitory concentration index (FICI) which is the sum of the fractional inhibitory concentration (FIC) of each drug [24]. The drug interaction was defined as the following: FICI ≤ 0.5, synergism; FICI > 0.5 to ≤ 4.0, indifference; FICI > 4.0, antagonism. The FICI values were calculated based on the MIC-2 endpoint for all drugs.

## Results

To our knowledge, no standard interpretive breakpoints are available for *in vitro* antifungal susceptibility testing against *Trichosporon* species. However, the breakpoints for *Candida* species have been used in the *in vitro* antifungal susceptibility testing against *Trichosporon* isolates previously [25,26]. Thus, the reference breakpoints for *Candida* species were cautiously used to interpret results obtained in this study. To evaluate drug interactions, we compared the MICs of each antifungal drug alone to the MICs of antifungal combinations with SRT and calculated the FICIs of different antifungal combination.

The MIC range, geometric mean (GM) and the MICs for 50% or 90% of the isolates (MIC_50_/MIC_90_) for *T*. *asahii* planktonic cells are present in Table 1. The *in vitro* antifungal susceptibility results by using both MIC-0 and MIC-2 endpoints on the anti-*T*. *asahii* activities of AMB and SRT are present in Table 2. The interactions of each antifungal combination against *T*. *asahii* planktonic cells are present in Table 3. The SMIC range, GM and the interactions of each antifungal combination against *T*. *asahii* biofilms are present in Table 4.

**Table 1:** MICs of antifungal drugs and sertraline against planktonic forms of 21 *T*. *asahii* isolates.

Drug combination	MIC (μg/ml)											
	Alone						In combination					
	SRT			antifungal drug			SRT			antifungal drug		
	MIC range	GM	MIC_50_/MIC_90_	MIC range	GM	MIC_50_/MIC_90_	MIC range	GM	MIC_50_/MIC_90_	MIC range	GM	MIC_50_/MIC_90_
SRT / FLC	4–8	5.560	4/8	1–16	2.875	2/4	1–4	1.696	2/4	0.25–1	0.348	0.25/0.5
SRT / ITC	4–8	5.560	4/8	0.25–1	0.484	0.5/1	0.5–4	1.141	1/2	0.062–0.25	0.138	0.125/0.25
SRT / VRC	4–8	5.560	4/8	0.031–0.25	0.071	0.0625/0.125	1–4	1.872	2/4	0.016–0.062	0.031	0.031/0.062
SRT / CAS	4–8	5.560	4/8	8–32	20.159	16/32	0.5–4	1.179	1/4	0.5–8	2.438	2/4
SRT / AMB	4–8	5.560	4/8	0.25–4	1.486	2/2	0.5–2	0.906	1/2	0.031–0.125	0.069	0.25/0.5

MIC, the lowest concentrations that causing a 50% reduction in turbidity compared to the growth control wells for all antifungal drugs and SRT; MIC_50_, the lowest concentrations that inhibiting 50% of the total *T*. *asahii* isolates; MIC_90_, the lowest concentrations that inhibiting 90% of the total *T*. *asahii* isolates; GM, the geometric means of MIC values; FLC, fluconazole; ITC, itraconazole; VRC, voriconazole; CAS, caspofungin; AMB, amphotericin B; SRT, sertraline

**Table 2:** MIC-0 and MIC-2 results of the anti-*T*. *asahii* susceptibility activity of AMB in combination with SRT.

Endpoints	MIC (μg/ml)											
	Alone						In combination					
	SRT			AMB			SRT			AMB		
	MIC range	GM	MIC_50_/MIC_90_	MIC range	GM	MIC_50_/MIC_90_	MIC range	GM	MIC_50_/MIC_90_	MIC range	GM	MIC_50_/MIC_90_
MIC-0	8–32	14.97	16/32	1–8	4.718	4/8	1–8	1.696	2/8	0.062–0.5	0.212	0.25/0.5
MIC-2	4–8	5.560	4/8	0.25–4	1.486	2/2	0.5–2	1.141	1/2	0.031–0.125	0.069	0.062/0.125

MIC-2, the lowest concentrations that causing a 50% reduction in turbidity compared to the growth control wells; MIC-0, the lowest concentrations that causing complete inhibition of growth; MIC_50_, the lowest concentrations that inhibiting 50% of the total *T*. *asahii* isolates; MIC_90_, the lowest concentrations that inhibiting 90% of the total *T*. *asahii* isolates; GM, the geometric means of MIC values; AMB, amphotericin B; SRT, sertraline

**Table 3:** Interactions of antifungal drugs with sertraline on antifungal activities against *T*. *asahii* planktonic cells.

Drug combination	FICI		Interactions					
			Synergy		Indifference		Antagonism	
	Range	GM	n	%	n	%	n	%
SRT / FLC	0.156–1.125	0.452	13	61.9	8	38.1	0	0
SRT / ITC	0.25–1.5	0.573	9	42.9	12	57.1	0	0
SRT / VRC	0.25–1.5	0.853	5	23.8	16	76.2	0	0
SRT / CAS	0.188–0.75	0.350	17	81.0	4	19.0	0	0
SRT / AMB	0.094–0.563	0.228	19	90.5	2	9.5	0	0

FICI, fractional inhibitory concentration index; FICI ≤ 0.5, synergy; FICI > 0.5–4, indifference; FICI > 4, antagonism; GM, the geometric means of FICI values; FLC, fluconazole; ITC, itraconazole; VRC, voriconazole; CAS, caspofungin; AMB, amphotericin B; SRT, sertraline

**Table 4:** SMICs of antifungal drugs and sertraline against biofilms of 21 *T*. *asahii* isolates and the interactions of antifungal drugs with sertraline.

Drug combination	SMIC (μg/ml)				FICI	Interactions				
	Alone		In combination		Range (GM)	Syn	Ind		Ant	
						n (%)	n (%)		n (%)	
	SRT	antifungal drug	SRT	antifungal drug						
	SMIC range (GM)	SMIC range (GM)	SMIC range (GM)	SMIC range (GM)						
SRT / FLC	16-32(23.776)	512–1024 (897.348)	4-16(7.246)	128-512(301.940)	0.25–1.5 (0.678)	6 (28.6)		15 (71.4)		0
SRT / ITC	16-32(23.776)	>1024(>1024)	8–32 (12.287)	256–1024 (420.010)	0.5–2 (0.985)	5 (23.8)		16 (76.2)		0
SRT / VRC	16-32(23.776)	>1024(>1024)	8–32 (17.092)	512->1024 (736.130)	0.75–3 (1.497)	1 (4.8)		20 (95.2)		0
SRT / CAS	16-32(23.776)	16–64 (29.956)	2–16 (6.563)	2–32 (9.7520)	0.25–1 (0.527)	10 (47.6)		11 (52.4)		0
SRT / AMB	16-32(23.776)	128–1024 (603.870)	4–16 (7.489)	8–64 (23.004)	0.133–1.125 (0.384)	17 (81.0)		4 (19.0)		0

SMIC, the sessile minimum inhibitory concentrations capable of decreasing 50% in absorbance compared to the growth control wells; FICI, fractional inhibitory concentration index; FICI ≤0.5, synergy; FICI > 0.5–4, indifference; FICI > 4, antagonism; GM, the geometric means of MIC and FICI values; Syn, a combination indicating synergistic interaction; Ind, a combination indicating indifferent interaction; Ant, a combination indicating antagonistic interaction; FLC, fluconazole; ITC, itraconazole; VRC, voriconazole; CAS, caspofungin; AMB, amphotericin B; SRT, sertraline

When tested alone, the lowest MICs were obtained for VRC (MICs 0.031–0.25 μg/ml; GM, 0.071 μg/ml) against *T*. *asahii* planktonic cells, followed by ITC (MICs 0.25–1 μg/ml; GM, 0.484 μg/ml), FLC (MICs 1–16 μg/ml; GM, 2.875 μg/ml), AMB (MICs 1–8 μg/ml; GM, 4.718 μg/ml) and CAS (MICs 8–32 μg/ml; GM, 20.159 μg/ml). The MICs of VRC for all isolates were < 1 μg/ml and the MIC_50_ and MIC_90_ were both < 1 μg/ml. The MICs of FLC for all isolates were ≤ 16 μg/ml and the MIC_50_ and MIC_90_ of FLC were both ≤ 8 μg/ml. The MICs of ITC for all isolates were ≥0.25 μg/ml. The MIC_50_ and MIC_90_ of ITC were 0.5 μg/ml and 1 μg/ml, respectively. 81% of *T*. *asahii* isolates (17/21) showed MICs ≤ 0.5 μg/ml to ITC and 19% isolates (4/21) showed MICs ≥1 μg/ml. The MICs of CAS for all isolates were ≥ 8 μg/ml and the MIC_50_ and MIC_90_ were both higher than 16 μg/ml.

The MIC-2 and MIC-0 ranges for SRT were 4–8 μg/ml and 8–32 μg/ml, respectively. The MIC_50_/MIC_90_ for SRT by using MIC-2 endpoint and MIC-0 endpoint were 4/8 μg/ml and 16/32 μg/ml, respectively. Using MIC-0 endpoint, 95% (20/21) of *T*. *asahii* isolates showed MICs ≥ 2 μg/ml to AMB, the MIC_50_ and MIC_90_ to AMB were both ≥ 4 μg/ml. Using MIC-2 endpoint, 85.7% (18/21) of *T*. *asahii* isolates showed MICs ≥ 1 μg/ml to AMB and 66.7% (14/21) of *T*. *asahii* isolates showed MICs ≥ 2 μg/ml, the MIC_50_ and MIC_90_ to AMB were both 2 μg/ml.

For the antifungal combinations susceptibility testing against *T*. *asahii* planktonic cells, the SRT/AMB combination showed the highest percentage of synergistic effects (90.5%; FICI, 0.094–0.563). The MIC-2 ranges obviously decreased from 4–8 μg/ml to 0.5–2 μg/ml for SRT and from 0.25–4 μg/ml to 0.031–0.125 μg/ml for AMB. In contrast, the MIC-0 ranges obviously decreased from 16–32 μg/ml to 2–8 μg/ml for SRT and from 1–8 μg/ml to 0.062–0.5 μg/ml for AMB. The MIC_50_ and MIC_90_ using MIC-0 endpoint obviously decreased from 4–8 μg/ml to 1–2 μg/ml for SRT and from 4–8 μg/ml to 0.25–0.5 μg/ml for AMB. The SRT/CAS combination (81.0%; FICI, 0.188–0.75) and the SRT/FLC combination (61.9%; FICI, 0.156–1.125) also showed obvious synergistic effects. The combinations of SRT/ITC (57.1%) and SRT/VRC (76.2%) yielded mainly indifferent interactions. No antagonistic interaction was observed in any of the drug combinations against *T*. *asahii* planktonic cells.

The results of our *in vitro* anti-biofilms susceptibility testing showed that the SMICs of most antifungal drugs against *T*. *asahii* biofilms increased up to 1000 times compared to the MICs against planktonic cells except for CAS, whose SMICs increased only 2–4 folds. The SMICs (16–32 μg/ml) of SRT against *T*. *asahii* biofilms increased 4 folds compared to the MICs (MIC-2, 4–8 μg/ml) against planktonic cells, which indicated the decreased susceptibility of SRT to *T*. *asahii* biofilms.

For the antifungal combinations susceptibility testing against *T*. *asahii* biofilms, the SRT/AMB combination also showed the highest percentage of synergistic effects (81.0%; FICI, 0.094–0.563) and the SMICs obviously decreased from 16–32 μg/ml to 4–16 μg/ml for SRT and from 128–1024 μg/ml to 8–64 μg/ml for AMB. The SRT/CAS (47.6%) combination yielded relative lower percentage of synergistic interactions. The combinations of SRT/FLC (71.4%), SRT/ITC (76.2%), and SRT/VRC (95.2%) yielded mainly indifferent interactions. No antagonistic interaction was observed in any of the drug combinations against *T*. *asahii* biofilms.

## Discussion

*T*. *asahii* is the major pathogen of invasive trichosporonosis which occurred mainly in immunocompromised patients. Compared to the low incidence rates of invasive trichosporonosis, invasive *Trichosporon* infections leads to high mortality rates up to 80% despite treated with antifungal drugs [1].

To date, treatment of invasive trichosporonosis remains a challenge. Most of the *in vitro* antifungal susceptibility tests demonstrated high MICs of AMB and echinocandins to *T*. *asahii* and indicated drug resistance. In contrast, azoles antifungal drugs, especially the newer triazoles, are the primary drug class for the treatment of invasive trichosporonosis based on available data. However, decreases of the susceptibility of *T*. *asahii* to azoles have been reported, including the newer triazoles [7,8].

As expected, VRC (MICs, 0.031–0.25 μg/ml) was the most effective drug against *T*. *asahii* planktonic cells in this study. No significant high MICs of three azoles were observed against most *T*. *asahii* isolates in this study. ITC (MICs, 0.25–1 μg/ml) and FLC (MICs, 1–16 μg/ml) were still sensitive to most *T*. *asahii* isolates. All tested *T*. *asahii* isolates were resistant to AMB (MICs ≥ 2 μg/ml) and CAS (MICs ≥ 8 μg/ml). Our results were in agreement with previous data in China of the *in vitro* antifungal susceptibility of VRC, CAS and AMB against clinical *T*. *asahii* isolates [27].

Previous antifungal susceptibility assay demonstrated a remarkable rise in the sessile MICs of azoles against *T*. *asahii* biofilms (SMIC>1024 μg/ml) compared to the MICs of planktonic cells and *T*. *asahii* biofilms were up to 16000 times more resistant to VRC than planktonic cells [28]. In agreement with previous reports, *T*. *asahii* biofilms were resistant to all three azoles tested in this study, as the SMICs were up to 1000 times higher than the MICs of *T*. *asahii* planktonic cells. *T*. *asahii* biofilms were more resistant to AMB than planktonic cells.

As is well known, *T*. *asahii* is intrinsic resistant to echinocandins [1,2]. However, CAS was observed to inhibit *T*. *asahii* biofilms at the final concentrations from 16 to 64 μg/ml in this study, and the SMICs of CAS increased only 2–4 folds compared to the MICs of planktonic cells. The inhibitory effect on the synthesis of β-(1,3)-glucan of the fungal cell wall is believed to be one of the mechanisms of CAS to exert anti-biofilms effects against *C*. *albicans* biofilms, since β-(1,3)-glucan is considered to be a major component of fungal biofilms [29,30]. Thus, the inhibitory effect on the synthesis of β-(1,3)-glucan may also account for the anti-biofilms effects of CAS against *T*. *asahii* in this study. The possible inhibitory effects of CAS against *T*. *asahii* biofilms needs further studies to be validated.

Based on a review of 185 reported cases from 1975 to 2014, *Trichosporon* fungemia, including catheter-related fungemia, represents the main type of invasive *Trichosporon* infection [2]. However, disseminated trichosporonosis can involve most human organs and results in pneumonia, endocarditis, brain abscess, meningitis, arthritis, esophagitis, lymphadenopathy, liver infection, splenic abscess, uterine infection and soft tissue infection [1]. Invasive trichosporonosis are usually associated with the use of medical implanted devices [1,2]. Peritoneal dialysis can cause fungal peritonitis due to *Trichosporon* species [1]. Endocarditis due to *Trichosporon* spp. in cardiac valve replacement patients has been increasingly reported [1]. Urinary tract infections and renal dysfunction caused by *Trichosporon* spp. have also been reported, especially in patients with vesical catheterization [1]. Removal of infected catheters may increase the efficacy of antifungal drugs and improve clinical outcomes. Unfortunately, most severe patients are catheter-dependent and it is a life-threaten matter to remove medical implanted catheters [31]. Thus, the development of new antifungal agents with antifungal activity against *T*. *asahii* biolfilms is necessary.

SRT, a commonly prescribed psychotropic drug, was selected as a potential antifungal agent against *T*. *asahii* in this study based on its reported *in vitro* and *in vivo* fungicidal activity, low toxicity and lack of drug interactions [14–22,32]. SRT was firstly reported to show antifungal activity against *Candida* species in 2001 [14]. Lass-Flörl et al found that patients with recurrent vulvovaginal candidiasis were cured when treated with SRT for accompanying premenstrual dysphoric disorder [14]. From then on, the antifungal activities of SRT against *Candida* spp., *Aspergillus* spp. and *Cryptococcus* spp. have been extensively discussed [14–21]. The antifungal activity of SRT against *C*. *neoformans* has also been demonstrated in animal model studies [16,17]. SRT was demonstrated to reduce fungal burden in the brain, kidney and spleen in murine models of systemic cryptococcosis at clinically relevant concentrations [16,17]. More important, the antifungal effect of SRT against *Cryptococcus* infection has been demonstrated clinically [22]. Thus, SRT was speculated to have similar antifungal effect against *T*. *asahii*, which is phylogenetically closed to *C*. *neoformans*. Considering the important role of fungal biofilms in drug-resistance, the *in vitro* antifungal activities of SRT against both *T*. *asahii* planktonic cells and biofilms were evaluated in this study.

Our study demonstrated that SRT was fungicidal in high concentrations (MIC-0, 8–32 μg/ml). The MIC_90_ for SRT by using both MIC-2 and MIC-0 endpoints indicated that SRT could inhibit 90% of the total *T*. *asahii* isolates in concentration of 8 μg/ml and kill 90% of the total isolates in concentration of 32 μg/ml. The SMICs (16–32 μg/ml) of SRT demonstrated its inhibitory activity against *T*. *asahii* biofilms in concentrations higher than 16 μg/ml. Our results demonstrated that SRT exhibited fungicidal activity against *T*. *asahii* planktonic cells and inhibitory activity against *T*. *asahii* biofilms in relative high concentrations.

To evaluate the clinical therapeutic potential of SRT on invasive *T*. *asahii* infections, we compared the pharmacokinetic data of SRT with our *in vitro* antifungal susceptibility data. The MICs (MIC-2 ranges, 4–8 μg/ml; MIC-0 ranges, 8–32 μg/ml) against *T*. *asahii* are much higher than the reported blood concentrations of SRT (55–250 ng/ml) [13,33]. Pharmacokinetic studies of SRT also demonstrated that the concentrations of SRT in the brain were 20–50 times higher than blood concentrations [33]. Furthermore, the concentrations of SRT in the eyes, heart, lung, spleen, liver, kidney, stomach, small intestine, muscle and skin were also demonstrated to be much higher than the blood concentrations, although the organ/blood concentration ratios for these organs have not been assayed statistically [33,34]. Thus, the much higher concentrations in tissues and organs of SRT may be beneficial for treating disseminated trichosporonosis since *T*. *asahii* often disseminated to most organs of patients [1].

Although it is difficult to achieve high blood concentrations of SRT administrated orally, it is possible that the high concentrations may be attainable in catheters by way of intra-luminal lock therapy. Antifungal lock therapy is to use high local concentrations of antimicrobial agents within an infected catheter in attempt to sterilize the catheters [35]. The therapeutic potential of ethanol as lock therapy against *T*. *asahii* infection has been demonstrated by our research group previously [36]. Based on the anti-biofilm activity of SRT against *T*. *asahii* observed in this study, SRT may be used as a lock strategy with high local concentrations acting on infected catheters and may facilitate the clearance of *T*. *asahii* biofilms and improve clinical outcomes.

In addition to the antifungal activity of SRT alone, it has also been demonstrated that SRT exhibited *in vitro* synergistic effects in combination with antifungal drugs against *Aspergillus* spp. and *C*. *neoformans*. SRT was demonstrated to enhance the activity of AMB against *Aspergillus* spp. [19] and was also demonstrated to exhibit *in vitro* and *in vivo* synergistic effect in combination with FLC against *C*. *neoformans* [16,20]. SRT also exhibited *in vitro* synergistic effects combined with AMB against *C*. *neoformans* in another study [21]. Based on these previous studies, the possible synergistic effect of SRT in combination with antifungal drugs against both *T*. *asahii* planktonic cells and biofilms were further evaluated in this study.

Our results demonstrated that SRT indeed exhibited synergistic effects when combined with AMB, CAS or FLC against *T*. *asahii* planktonic cells. The combinations of SRT-AMB (90.5%), SRT-CAS (81.0%) and SRT-FLC (61.9%) yielded potent synergistic effects. In our anti-biolilms combinations study, the combination of SRT-AMB also showed the highest percentage of synergistic effects (81.0%). In contrast, SRT exhibited mostly indifferent interactions in combinations with three azoles. The SRT-CAS combination (47.6%) yielded relative lower synergistic effects against *T*. *asahii* biofilms compared to that of SRT-AMB.

Our antifungal combinations study highlights the therapeutic potential of SRT-AMB combination for *T*. *asahii* infection, since the SRT-AMB combination yielded highest percentage of synergistic effects against both *T*. *asahii* planktonic cells and biofilms. As is well known, AMB is a fungicidal drug with high toxic effect. Based on our results and previous data [14–18], SRT is also fungicidal. Thus, the SRT-AMB combination therapy may result a better therapeutic efficacy with reduced toxicity of AMB. Furthermore, The SRT-AMB combination may be beneficial for reducing the emergence of drug-resistance. Thus, the anti-biofilms effect of SRT alone and SRT-AMB combination on *T*. *asahii* highlights the potential utility of SRT or SRT-AMB combination on invasive *T*. *asahii* infections, especially suitable for the patients with medical implanted devices.

The antifungal mechanisms of SRT are not investigated in this study. However, some possible antifungal mechanisms of SRT have been discussed by different research group. A genetic study suggests that SRT may exert antifungal effect by perturbing translation and inhibiting protein synthesis of fungi [16]. Rainey et al. demonstrated that SRT may exhibit antifungal activity by targeting intracellular vesiculogenic phospholipid membranes in fungi [37]. Another study demonstrated that SRT can perturb membrane permeability and inhibit sphingolipid biosynthesis in fungi [38]. These reported antifungal mechanisms of SRT may also account for the antifungal activity against *T*. *asahii* in this study.

The antifungal synergistic mechanisms have also been discussed previously [22]. The different antifungal mechanisms of FLC (inhibiting ergosterol synthesis) and SRT (inhibiting mRNA translation into protein synthesis) may account for their synergistic antifungal effects [22]. As is well known, AMB exerts antifungal effect by binding with ergosterol, forming channels in fungal cell membranes that cause rapid leakage of cell contents and subsequent fungal cell death. The different antifungal mechanisms of AMB and SRT may also account for their synergistic anti-*T*. *asahii* effects observed in this study.

In summary, our study demonstrates the *in vitro* antifungal activities of SRT on both *T*. *asahii* planktonic cells and biofilms and highlights the therapeutic potential of SRT against invasive *T*. *asahii* infections, especially suitable for the patients with catheter-related fungal infections. The use of the SRT-AMB combination therapy may be advantageous in treating *T*. *asahii* infection based on their obviously synergistic effects. The anti-biofilms activity of SRT against *T*. *asahii* may be helpful to control biofilms-related fungal infection, not only for *T*. *asahii*, but also for other pathogenic fungi (such as *C*. *neoformans*). Considering the clinical *T*. *asahii* isolates used in this study were mainly from China, the *in vitro* fungicidal activity and anti-biofilms activity of SRT against *T*. *asahii* should be confirmed by researchers outside China. Lack of *in vivo* anti-*T*. *asahii* data of SRT is a limitation of this study. Further animal models and clinical trials are needed to validate the correlation of our findings. The precise antifungal mechanisms of SRT are also worthy to be investigated.

## Supporting Information

S1 Table MICs, SMICs and FICIs of Fluconazole (FLC), itraconazole (ITC), voriconazole (VRC), caspofungin (CAS) or amphotericin B (AMB) in combination with sertraline (SRT) against all of 21 *T*. *asahii* isolates. MICs, the minimum inhibitory concentrations. SMICs, the sessile minimum inhibitory concentrations. FICI, the fractional inhibitory concentration index.
(XLS)
